# Machine learning-based obesity classification considering 3D body scanner measurements

**DOI:** 10.1038/s41598-023-30434-0

**Published:** 2023-02-26

**Authors:** Seungjin Jeon, Minji Kim, Jiwun Yoon, Sangyong Lee, Sekyoung Youm

**Affiliations:** 1 Department of Industrial and Systems Engineering, Dongguk University, 30 Pildong-ro 1-gil, Jung-gu, Seoul, 04620 Republic of Korea; 2 Department of Physical Education, Korea National Sport University, Seoul, Republic of Korea

**Keywords:** Health care, Health services, Weight management

## Abstract

Obesity can cause various diseases and is a serious health concern. BMI, which is currently the popular measure for judging obesity, does not accurately classify obesity; it reflects the height and weight but ignores the characteristics of an individual’s body type. In order to overcome the limitations of classifying obesity using BMI, we considered 3-dimensional (3D) measurements of the human body. The scope of our study was limited to Korean subjects. In order to expand 3D body scan data clinically, 3D body scans, Dual-energy X-ray absorptiometry, and Bioelectrical Impedance Analysis data was collected pairwise for 160 Korean subjects. A machine learning-based obesity classification framework using 3D body scan data was designed, validated through Accuracy, Recall, Precision, and F1 score, and compared with BMI and BIA. In a test dataset of 40 people, BMI had the following values: Accuracy: 0.529, Recall: 0.472, Precision: 0.458, and F1 score: 0.462, while BIA had the following values: Accuracy: 0.752, Recall: 0.742, Precision: 0.751, and F1 score: 0.739. Our proposed model had the following values: Accuracy: 0.800, Recall: 0.767, Precision: 0.842, and F1 score: 0.792. Thus, our accuracy was higher than BMI as well as BIA. Our model can be used for obesity management through 3D body scans.

## Introduction

Grading a patient’s obesity level is an important activity in healthcare^1^. Obesity acts as a risk factor for various diseases, including chronic diseases, type-2 diabetes, heart disease, and certain cancers^2–7^. Knowing that you are obese can motivate you to manage your weight^8,9^. In addition, intentional weight management can lower the risk of poor health and is associated with the benefit of a lower risk of disease^10^. However, Body Mass Index (BMI), which represents the criteria for obesity defined by the World Health Organization (WHO), does not accurately classify obesity as it does not sufficiently reflect body-type factors^11–13^. Nutrition varies between regions and body types^14,15^. Reflecting the body-type factor requires anthropometric measurements. However, traditional anthropometric methods are impractical because such measuring requires trained experts. As an alternative, research on using a 3D scanner for human body measurement is being actively conducted^16–27^ as it is a less-invasive method than traditional anthropometric measurements. Computed Tomography (CT) or Dual-energy X-ray absorptiometry (DXA), which is the gold standard for measuring human body fat percent (bf%), involve the risk of exposure to radiation when frequent measurements are taken. Unlike CT or DXA, 3D scanners do not expose the human body to radiation^26^. In addition, health risks can be analyzed or predicted through various measures, not just a single one. Therefore, in this study, 3D body scan data and DXA data were collected in pairs for Koreans, and obesity classification was performed using anthropometric values obtained from the 3D body scan data.

Löffler-Wirth et al. classified the body types of residents in Leipzig, Germany using a 3D scanner^17^. Body measurements were extracted from 8499 people through a 3D scanner. After dividing this by height, the body types were grouped through Self-organizing Map (SOM). Clustering body types using a Machine Learning methodology through large-scale experiments is meaningful, but it is not scalable because it cannot be paired with clinical information such as DXA. Pleuss et al.^25^ conducted a sampling study to supplement Löffler-Wirth et al. but the number of samples was quite small. In Ng et al. and Bennett et al. 3D scanner data and DXA showed a strong correlation through statistical analysis; the relationship can be explained but it is difficult to estimate bf% through various factors of the human body^16,27^. Some studies used machine learning; Harty et al.^21^ developed a new bf% estimation equation based on 3D human body data and 4C model anthropometric data. Using a decision tree model is useful in that important factors and the criteria necessary for formulas can be distinguished. Lu et al.^20^ proposed a methodology to predict bf% through a machine learning-based framework after extracting features from 3D body data obtained using a 3D scanner. Although it showed higher accuracy than BMI and BOD POD, the experiment was conducted on relatively few subjects, i.e., 50 men. Although active research is underway on DXA and 3D body scan data, not many machine learning models have been studied.

Most obese people do not perceive themselves as obese^28,29^, which may hinder public health initiatives^30^. Knowing one's obesity group can serve as a prerequisite for behavior change for health^31^ and will be helpful for health management and disease prevention.

The purpose of our study is to develop a machine learning framework to classify obesity among Koreans based on the bf% of DXA—considered the gold standard—using body measurements extracted from 3D scanners. By selecting input features through a Genetic Algorithm, we not only improve the performance of the Machine Learning model but also observe the selected input features to help in healthcare.

## Method

### Materials

**Figure 1:**
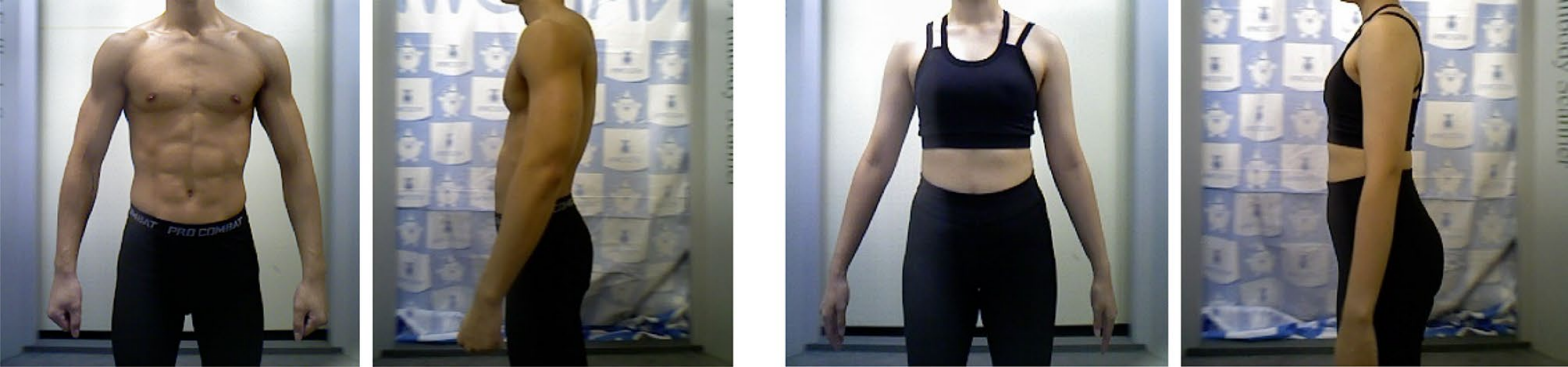
Subjects’ attire and posture.

The collection and use of data used in this study and ethical review were approved by the Institutional Review Board of Korea National Sport University (20220411-021). All methods were carried out in accordance with relevant guidelines and regulations. Informed consent was obtained from all subjects and/or legal guardians regarding including their information/images in an online open-access publication and paper. We confirmed that informed consent was obtained from all subjects (for participation). This experiment faithfully followed the strict regulations and guidelines of the Institutional Review Board. The dataset used in this study was collected between 2022–04–11 and 2022–06–30. Recruitment was based on BMI to have a BMI distribution similar to Size Korea. There were a total of 160 subjects: 73 women and 87 men. As shown in Fig. 1, the men wore tight-fitting bottoms and swimming caps and the women wore tight-fitting tops and bottoms and swimming caps. The subjects were measured using a 3D scanner, DXA, and BIA. Through this, a total of 160 3D body data, DXA data, and BIA data were obtained. Statistics for the collected data are presented in Table 1.

**Table 1:** Collected data statistics.

Gender	Statistics	Age	Height (cm)	Weight (kg)	BMI (kg/m^2^)	DXA BF(%)	BIA BF(%)
Male (87)	Average	24.07	178.10	77.92	24.64	20.13	18.45
	Std	4.25	5.37	13.52	4.00	8.70	7.42
	Min	20.00	165.55	54.30	16.22	5.50	5.40
	Max	39.00	191.83	120.90	37.61	37.40	37.00
Female (73)	Average	24.14	165.63	57.01	20.74	27.55	25.83
	Std	4.54	4.96	10.13	3.34	6.94	6.29
	Min	20.00	155.87	40.70	15.70	6.70	11.70
	Max	37.00	173.99	89.60	32.25	46.00	45.70

First, we verified whether our 160 data were a fair representation of general Korean anthropometric data. The 8th Anthropometric Data collected between 2020 and 2021 by Size Korea was used for validation^32^. As our main participants were in their 20 s and 30 s, we used the data of 1547 women and 1306 men in 20 s and 30 s from amongst the 8th Anthropometric Data for accurate comparison. In order to verify whether the anthropometric characteristics of Koreans can be viably represented, a Kolmogorov–Smirnov (KS) test was conducted to confirm that the two sample distributions were identical. This test is useful for determining the difference in variance between two samples. In the case of men, the *p* value of the KS test came out as 0.926, which is valid at the significance level of 0.01. In the case of women, the *p* value of the KS test was 0.052, which is valid at the significance level of 0.01. And a t-test was conducted to compare the average BMI within the data we collected with the average BMI within the Size Korea data. For men, the t-test result was a *p* value of 0.5807, at a significance level of 0.01, so we hypothesized that there is no difference between the average BMI of Korean men in 20 s and 30 s and the average BMI of men we collected. Similarly, for women, the t-test result was a *p* value of 0.0532, and at a significance level of 0.01, so we hypothesized that there is no difference between the average BMI of Korean women in 20 s and 30 s and the average BMI of women we collected. Thus, we verified that the data we collected was representative of Koreans in 20 s and 30 s.

### 3D scanner

**Figure 2:**
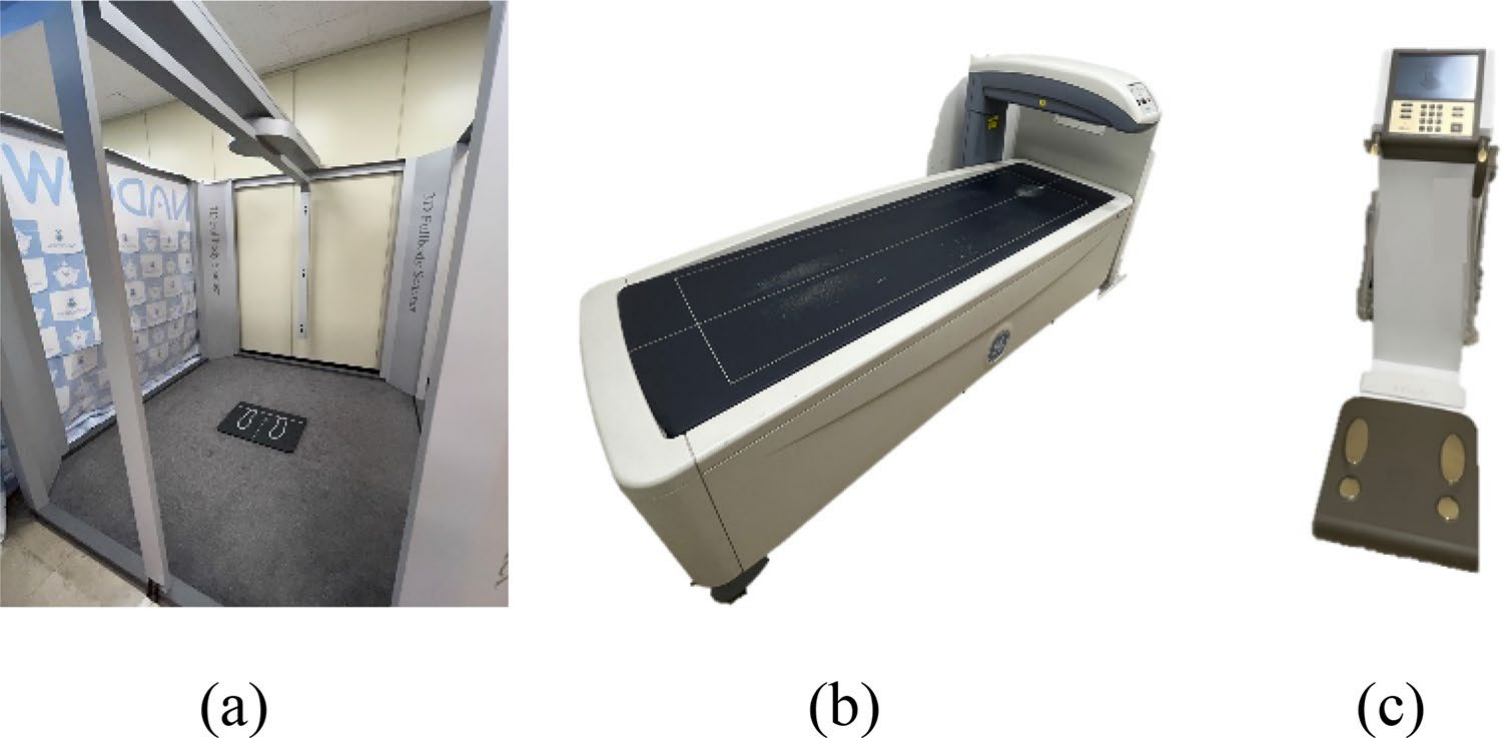
Equipment used in the experiment. (**a**) 3D scanner (PFS-304 of PMT), (**b**) DXA (Lunar of GE), (**c**) BIA (Inbody770).

The 3D scanner was a PFS-304A model (PMT innovation company, Gyeonggi-do, Korea, PFS software ver. 1.3). Figure 2a shows the 3D scanner used in this study, wherein the camera module rotates 360° through a motor mounted on the top when it scans and subjects can be measured in a stationary position. The subjects’ postures were measured by taking the A-pose recommended by ISO-7250^33^. If the A-pose is not taken (e.g., anthropometry is not performed when the arm is close to or attached to the body), a blind spot is formed and the correct mesh shape is not created. As shown in Fig. 2a, the measurements of the 3D scanner were taken in an indoor lighting environment, and they are summarized in Table 2.

**Table 2:** Measurement of 3D mesh data.

Value	description	Value	description
WCR	Waist-to-chest ratio	Thick thing cir	Circumference of the thickest part of the innermost thigh
WHR	Waist-to-hip ratio	Middle thigh cir	Circumference of the middle of the thigh
WHtR	Waist-to-height ratio	Knee cir	Horizontal circumference through the midpoint of the kneecap
THR	Thigh-to-height ratio	Calf cir	Circumference of the most convex part of the calf
Height of the back of the neck	Height from floor to the back of the neck in a standing position	Arm cir	Circumference of the thickest part of the arm
Shoulder height	Height from floor to the shoulders in a standing position	CS. area of the back of the neck	Cross-sectional area of the back of the neck
Chest height	In a standing position, the height from floor to the top of the nipples and the beginning of the chest	Shoulder CS	Cross-sectional area of ​​the shoulder
(breast) Chest height	Height from floor to nipple in standing position	Chest area	Cross-sectional area of ​​the part passing through the midpoint of the sternum
Waist height	Height from floor to the point in front of the waist in a standing position	(breast) Chest area	Cross-sectional area of ​​the part passing through the nipple point
Navel height	Height from the standing position to the navel	Waist CS	Cross-sectional area of ​​the part passing through the front of the waist, the side of the waist, and the back of the waist
Height below the navel	The height from the standing position to the iliac bone below the navel	Navel waist CS	Cross-sectional area of ​​the part passing through navel point, navel level, waist point, navel level, back point
Hip height	Height from floor to hip protrusion in standing position	Area below the navel	Cross-sectional area of ​​the part that passes through iliac crest below navel
Groin height	Vertical height from floor to groin (actual leg length)	Hip CS	Cross-sectional area of ​​the part passing through the buttock protrusion
Thick thigh height	Height from floor to the thickest part of the thigh	Groin area	Cross-sectional area of ​​​​the groin
Midthigh height	Height from floor to mid-thigh	Thick thigh area	Cross-sectional area of ​​the thickest part of the innermost thigh
Knee height	Vertical height from floor to the top of the shinbone	Meddle thigh area	Cross-sectional area of ​​​​the middle part of the thigh
Calf height	Height from floor to the point of the thickest part of the calf	Knee CS	Cross-sectional area passing through the midpoint of the kneecap
Neck cir	Circumference passing under the back of neck and under the shield cartilage	Calf CS	Cross-sectional area of ​​the most convex part of the calf
Shoulder cir	Circumference from the end of the shoulder to the end of the shoulder opposite the back of the neck	Total volume	Volume of total body
Cir. of the chest	Horizontal circumference through the midpoint of the sternum	Shoulder volume	Volume of shoulder
(breast)Chest cir	Horizontal circumference through nipple point	Chest volume	Volume of chest
Waist cir	Horizontal circumference passing through the point in front of the waist, the point in the side of the waist, and the point in the back of the waist	Epigastric volume	Volume of epigastrium
Navel waist cir	Horizontal circumference passing through navel point, navel level, waist point, navel level, back point	Lower abdominal volume	Volume of lower abdomen
Below navel cir	Horizontal circumference through the iliac crest below the navel	Thigh volume	Volume of thigh
Hip cir	Horizontal circumference through the buttock protrusion	Calf volume	Volume of calf
Groin cir	Groin circumference	Abdominal volumetric	Volume of abdominal

### Dual-energy X-ray absorptiometry (DXA)

Lunar (GE Healthcare, Madison Wisconsin, America, EnCore software ver. 13.60.03) in Fig. 2b was used for DXA. DXA is a body component-measuring instrument that measures body fat, lean body mass, and bone mass and has long been regarded as the gold standard for measuring body components. The DXA device was handled and measured by an expert; during measurement, the subject maintained an immobile posture while lying down, and the following body parts were examined: arm (left, right), leg (left, right), trunk (left, right), Android, and Gynoid. BMD, BMC, fat%, fat(g), and lean(g) were obtained through DXA for each body part. Here, BMD represents bone density and BMC represents bone mineral mass. Fat% represents fat percentage, fat (g) represents fat weight, and lean (g) represents weight excluding fat. This study classified obesity in individuals based on the total body fat% (bf%) according to DXA.

### Bioelectrical impedance analysis (BIA)

BIA is a method of measuring body composition through the impedance difference between body fat and lean body mass^34^. As shown in Fig. 2c, Inbody770 was used as the measuring device and the subjects were measured in the same clothing and immobile posture as in the previous experiments. Total Body Water (TBW), IntraCellular Water (ICW), ExtraCellular Water (ECW), protein, minerals, Body Fat percentage (bf%), and Fat-free Mass (FFM) were obtained through BIA. Among them, bf% was used for comparison using the methodology proposed in this study. The obesity group of BIA was divided using the bf% criteria presented in Table 3.

**Table 3:** Obesity class standard cutoff.

Index	Sex	Underweight	Normal	Overweight	Obesity
BMI (kg/m^2^)	Male/Female	BMI < 18.5	18.5 < BMI < 23	23 ≤ BMI < 25	25 ≤ BMI
BIA, DXA	Male	BF% < 10	10 ≤ BF% ≤ 20	20 < BF% ≤ 25	25 < BF%
	Female	BF% < 20	20 ≤ BF% ≤ 28	28 < BF% ≤ 35	35 < BF%

### Data preprocessing

**Figure 3:**
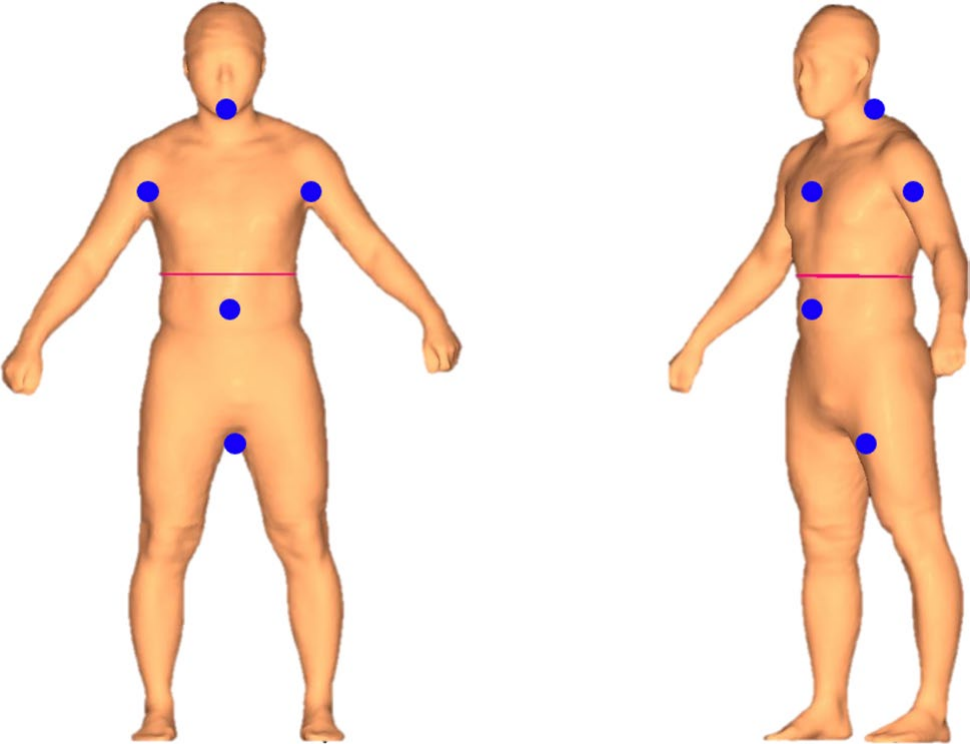
Sample of the 3D mesh data and standard landmarks for measurement.

Data obtained through DXA and BIA only used bf%. Based on the bf% obtained from the DXA of the test subjects in the experiment, the obesity class label was derived as per the cutoff in Table 3. The bf% standard cutoff for Obesity was set in accordance with the WHO^35^ classification, and the standard cutoffs from Lobman et al. were used for the rest, namely underweight, normal, and overweight^36^. Currently, there is no clear obesity category for DXA^37^, but the cutoff for obesity was based on 25%(bf%) for men and 35%(bf%) for women by the Korean Society for Obesity, and the rest of the groups were classified by reference to McArdle and Chang^38,39^. Therefore, subjects were labeled into four groups: underweight, normal, overweight, and obese. The distribution results of labeling based on bf% measured by DXA Table 3 are depicted in Table 4. As shown in Fig. 3, the data obtained from the 3D scanner extracted body measurements from the mesh data based on five landmarks: the back of the neck, the umbilicus, the groin, and the armpit (left, right).

**Table 4:** Subject class distribution.

Train set			
Class	Male	Female	Total
Underweight	8	4	12
Normal	23	23	46
Overweight	12	12	24
Obese	25	13	38

Test set			
Class	Male	Female	Total
Underweight	3	3	6
Normal	6	8	14
Overweight	5	6	11
Obese	5	4	9

### Framework

**Figure 4:**
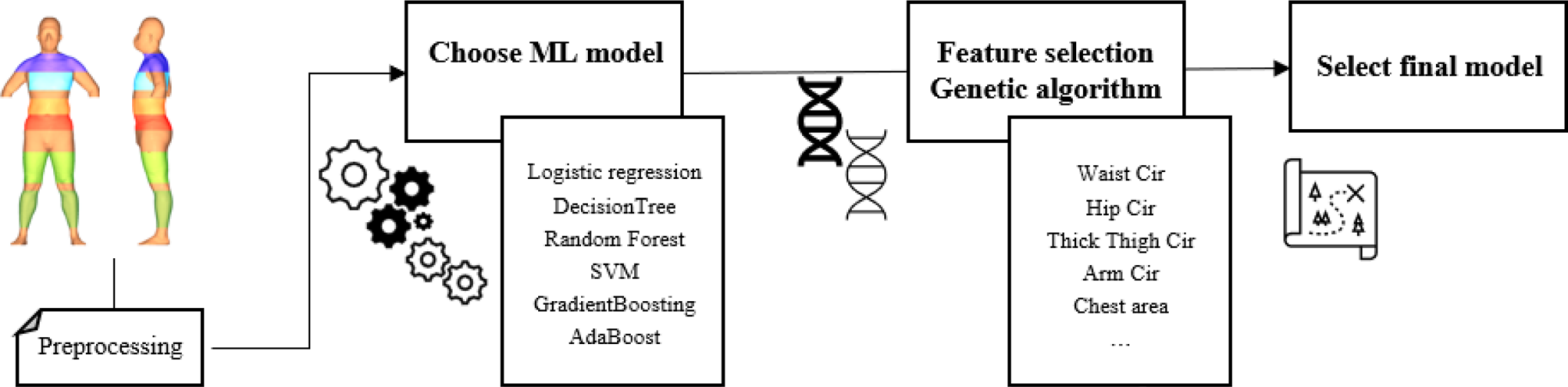
Overall framework of this study.

We propose a machine learning-based methodology to classify obesity groups. Figure 4 shows the proposed machine learning-based framework. Body measurements are first obtained from the 3D scanner, and then data preprocessing is performed by matching it with the bf% of DXA and labeling it. After that, the final model is selected through the process of “Choose ML model” and “Feature selection Genetic Algorithm.”

### Choose ML model

In data preprocessing, the data in the state of finished preprocessing the 3D body measurements and Sex are used as input values for the Logistic Regression^40^, Decision Tree^41^, Random Forest^42^, Support Vector Machine (SVM)^43^, Gradient Boosting^44^, and AdaBoost^45^ and fivefold cross validation is performed. It is divided into 120 training sets and 40 test sets. Among these models, Accuracy, F1, Recall, and Precision values were compared, and a model with good performance was selected and used as a classifier in the “Feature selection Genetic Algorithm” process. In Table 4, as the quantity of data is small and imbalances exist, the model is selected by referring to Precision, Recall, and F1 score values rather than simply using Accuracy. Accuracy is the ratio of correctly predicted numbers to the total number. Precision is the sum of true positives and false positives and the ratio of true positives. Recall is the sum of true positives and false negatives and the ratio of true positives. F1 score is the harmonic mean of Precision and Recall.

Logistic regression predicts the probability of occurrence by using a linear combination between variables of input data, and the result is classified into a specific class. This study used multiclass logistic regression with a cross-entropy function. Decision Tree is the most preferred machine learning model as an explanatory model. It outputs a class in which input data is classified based on input variables through a tree structure; it is a way to perform a query on a node and branching out. Its performance is not as good as other models, and it is vulnerable to overfitting. Random Forest is a machine learning model that uses a bundle of basic decision trees and averages them to compensate for performance. Through this, the performance can be generalized and made more robust against overfitting compared to a decision tree. Support Vector Machine determines the hyperplane to maximize the margin between support vectors; its purpose is to maximize the distance between various classes and to find a hyperplane that has a large difference from the training data to which the hyperplane is closest. The main idea of Gradient Boosting is to connect multiple non-deep decision trees, that is, weak learners. As basic trees can classify some data well, performance improves when trees are added. The loss function is defined and gradient descent is used to supplement the value to be classified by the next tree. AdaBoost stands for Adaptive Boosting. Unlike Gradient Boosting, this model is trained by adding weights to the classified samples. At this time, the learning model is created by adding weights to the next model in the sample that is poorly classified.

### Feature selection Genetic Algorithm

This process selects the input features of the previously selected machine learning model through a Genetic Algorithm. Selectively choosing the input features of the machine learning model not only improves the model’s performance but also identifies whether a specific value among the 3D body measurements in Table 2 affects the classification of obesity.

While selecting input features, finding the Global Optimum by comparing all sets of input features combinations is practically impossible. Therefore, a meta-heuristic algorithm approach was chosen to find an optimal solution close enough to the Global Optimum. Previous studies have demonstrated that the Genetic Algorithm is superior to other meta-heuristic algorithms in variable selection^46,47^. In this study, the Genetic Algorithm (GA) was used as a feature selection method. GA takes a meta-heuristic approach to solving complex problems through efficient trial and error^48^, hence mimicking Charles Darwin's theory of natural selection and mammalian reproduction. In this study, GA aims to find the best input feature through repeated generation reproduction. GA involves six steps. In Step 1, it initializes the combination of chromosomes, i.e., the initial input features, and sets the parameters. These parameters include population and mutation ratio, where population refers to the number of chromosomes in each generation, i.e., the number of combinations of input features. The mutation ratio refers to the ratio of gene mutations among all chromosomes; this corresponds to the ratio of selection of input features. We set the population to 100 and the mutation ratio to 20%. Step 2 involves learning each input feature in a Random Forest. In Step 3, fitness was evaluated for the chromosomes of each input feature and the fitness function was used to determine the accuracy. In Step 4, out of the current generation and current chromosomes, we selected excellent chromosomes with Accuracy. In this study, the top 80% were selected as excellent chromosomes. Step 5 involved generating next-generation chromosomes through crossover and mutation. In this case, crossover means mixing the selected adoptive parent chromosomes in half. We set this to stop when the 100^th^ generation was passed, and until then, it was set to return to Step 2 and repeat all intervening steps. In Step 6, we selected the final model, picking the model that generated the highest Accuracy.

**Figure 5:**
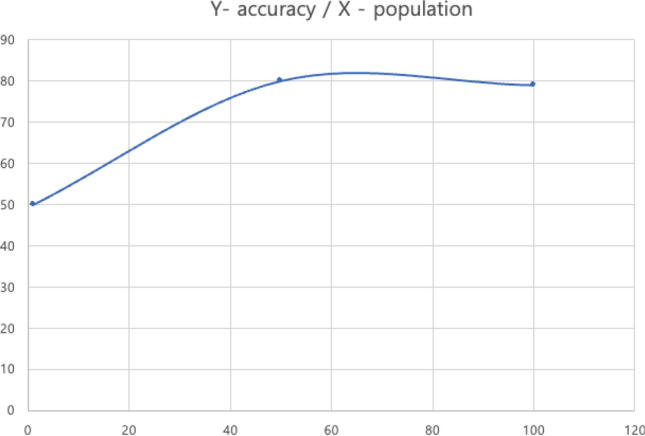
Accuracy flowchart by generation.

A total of 100 generations were generated and the input feature of the generation with the highest Accuracy, Recall, Precision, and F1 score was selected. Among the 100 generations, the generation with Accuracy, Recall, Precision, and F1 score of 0.8, 0.767, 0.842, and 0.792 was the highest and the corresponding input feature was selected as the final input feature. Accuracy reached 80% in the 50th epoch, after which it converged or even decreased. Figure 5 shows the flow of accuracy by generation. Table 5 presents the final selected features.

**Table 5:** Selected features of the “Feature selection Genetic Algorithm”.

Value	Value	Value
WCR	Hip cir	Hip CS
Groin height	Thick thing cir	Groin area
Thick thigh height	Middle thigh cir	Thick thigh area
Midthigh height	Arm cir	Calf CS
Knee height	Shoulder CS	Total volume
Calf height	Chest area	Shoulder volume
Shoulder cir	Navel waist CS	Thigh volume
(breast) Chest cir	Area below the navel	Sex

## Results

Accuracy, Recall, Precision, and F1 score were calculated using DXA as the Ground Truth for the reference group classification. BIA classified obesity based on the bf% obtained through Inbody770, and BMI classified obesity according to the WHO Asian standard cutoff^13^. The cutoff for BIA and BMI is listed in Table 3.

Table 6 shows the results of classification using the above models; among them, as its Accuracy, Recall, Precision, and F1 score were 0.725, 0.692, 0.661, and 0.78, i.e., all higher than those of other models, Random Forest was selected as the machine learning model in the process.

**Table 6:** Results of the choosing the ML model.

Rank	Classifier	Accuracy	Recall	Precision	F1 score
1	Random Forest	**0.725**	**0.661**	**0.780**	**0.692**
2	GradientBoosting	0.700	0.599	0.655	0.609
3	Logistic	0.500	0.515	0.443	0.462
4	SVM	0.650	0.477	0.383	0.412
5	DecisionTree	0.475	0.484	0.478	0.465
6	AdaBoost	0.425	0.358	0.431	0.356

Significant values are in bold.

The performance of the proposed approach is better than that using BMI. In Table 7, there is a difference of 0.271 in Accuracy from 0.529 to 0.8, 0.295 in Recall, 0.384 in Precision, and 0.33 in F1 score. This means that obesity can be classified more comprehensively by reflecting the various dimensions of the human body considering the body type rather than just the BMI, which classifies obesity through simple height and weight. Although BIA is widely used in body composition studies, concerns about its accuracy still exist^49,50^. As BIA assumes that the percentage of body water is approximately 73% and estimates it accordingly, low accuracy may ensue when the percentage of body water of an individual does not meet these conditions^51^. Furthermore, depending on the statistical model derived from a specific population, BIA may have differences in gender, age, ethnicity, and so on^50^. The Pearson’s correlation between bf% of DXA and bf% of BIA in our collected data was 0.95. When evaluated after the obesity classification presented in Table 3, there was a misclassification. BIA scored 0.752, 0.742, 0.751, and 0.739 in Accuracy, Recall, Precision, and F1 scores, respectively. The proposed obesity classification showed an improvement of 0.048, 0.025, 0.091, and 0.053 in Accuracy, Recall, Precision, and F1 scores, respectively, compared to BIA. This shows that the Accuracy, Recall, Precision, and F1 score are 0.075, 0.1, 0.106, and 0.062 higher, respectively, than in the Random Forest model without feature selection. The selected features affect the classification of obesity more than the unselected features. We also compared with the model provided by Tian et al.^52^. Denotes, Tian's model is a regression model, not a classification model, and we obtained results by dividing it by the criteria of the Table 3. The R^2^ and RMSE values of Tian's model were 0.56 and 5.74, respectively.

**Table 7:** Comparison between our method, BMI, BIA and Tian et al. within the test set.

Classifier	Accuracy	Recall	Precision	F1 score	R^2^/RMSE
Ours	**0.800**	**0.767**	**0.842**	**0.792**	
w/o GA	0.725	0.661	0.780	0.692	
BIA	0.752	0.742	0.751	0.739	
BMI	0.529	0.472	0.458	0.462	
Tian et.al	0.472	0.379	0.448	0.321	0.56/5.74

*w/o* without, *GA* genetic algorithm.

Significant values are in bold.

## Conclusion

We collected 3D body scans, DXA, and BIA data pairwise for Korean subjects and used this data to classify obesity in individuals. By using not only 3D body data but also DXA and BIA, we developed a technique for clinically clear obesity judgment that is expandable in terms of healthcare.

This study proposes a methodology for classifying obesity using various body measurements through a 3D scanner, unlike the BMI, which classifies obesity solely based on height and weight. The present study specifically considers Korean body types. The proposed methodology showed better performance in classifying obesity than the BMI through the machine learning methodology. It also showed better performance than BIA.

Pleuss et al. and Harty et al. conducted analysis using one or two machine learning models, but in this study, six machine learning models were compared to select a model suitable for obesity classification^21,25^. We performed feature selection through a Genetic Algorithm to identify the measurements of the human body that have an impact on determining obesity.

The proposed system showed superior performance over BMI and BIA. It can be used for long-term obesity healthcare monitoring by measuring one's body with a 3D scanner. Furthermore, as future healthcare is predicted to be Predictive, Preventive, Personalized, Participatory (4P)^53^, a system that classifies obesity as an indicator of health can be utilized as a new healthcare service that satisfies the 4P.

Our study has certain limitations. First, there are limits on data. The collected data included males and females in their 20 s and 30 s. As such, it is not common to all age groups. It does not reflect the adult group over 40 years of age. Furthermore, obesity was not evenly distributed, and there was a limited amount of data. Given that the measurement values were extracted and used from the mesh data generated by the 3D human body scanner, it is difficult to ensure that the 3D information was fully used. Second, there are spatial and cost limitations associated with 3D scanners that make them unpopular. Third, it provides users with categorical information rather than continuous information. It does not reflect the continuous variation of human.

We intend to collect continuous pair data as future work and conduct experiments in various age groups as well as compare between men and women in their 20 s and 30 s. Furthermore, we intend to conduct experiments on patients with specific diseases or disorders. By referring to previous studies^54^, we intend to consider using a three-dimensional representation such as curvature. In the Genetic Algorithm selection, selection methods such as elitism and tournament can be introduced to reduce computation complexity and optimize future research^55,56^. In order to reflect the continuous variability of human beings, we intend to introduce the regression methodology as a future study. We would like to observe the consistency of data obtained from different scanner devices and DXA as future research. We need to confirm this and do future work to validate the data and other models.

## Data Availability

The datasets generated and/or analyzed during the current study are not publicly available because they contain personal information but are available from the corresponding author upon reasonable request.
